# Supplementary material for: End Sequence Analysis Toolkit (ESAT) expands the extractable information from single-cell RNA-seq data
Source: Genome Res. 2016 Oct;26(10):1397–410. doi: 10.1101/gr.207902.116 (PMC5052061; doi:10.1101/gr.207902.116)
Supplement: Supplemental Material [file supp_gr.207902.116_Supp_Materials.tar.gz › ESAT-esat_v0.1/doc/html/classbroad_1_1core_1_1sequence_1_1_window_slider.html]

ESAT: broad.core.sequence.WindowSlider Class Reference


ESAT


- Main Page
- Related Pages
- Packages
- Classes
- Files

- Class List
- Class Index
- Class Hierarchy
- Class Members

All Classes Namespaces Files Functions Variables Enumerator Pages

- broad
- core
- sequence
- WindowSlider

Public Member Functions |
Static Public Member Functions |
List of all members

broad.core.sequence.WindowSlider Class Reference

Inheritance diagram for broad.core.sequence.WindowSlider:

[legend]

Collaboration diagram for broad.core.sequence.WindowSlider:

[legend]

|  |  |
| --- | --- |
| Public Member Functions | |
| boolean | hasNext () |
|  | |
| SequenceRegion | next () |
|  | |
| void | remove () |
|  | |

|  |  |
| --- | --- |
| Static Public Member Functions | |
| static WindowSlider | getSlider (Sequence seq, int windowSize, int overlap) |
|  | |
| static WindowSlider | getSlider (SequenceRegion seq, int windowSize, int overlap) |
|  | |

## Member Function Documentation

|  |  |  |  |
| --- | --- | --- | --- |
| static WindowSlider broad.core.sequence.WindowSlider.getSlider | ( | Sequence | *seq*, |
|  |  | int | *windowSize*, |
|  |  | int | *overlap* |
|  | ) |  |  |

static

|  |  |  |  |
| --- | --- | --- | --- |
| static WindowSlider broad.core.sequence.WindowSlider.getSlider | ( | SequenceRegion | *seq*, |
|  |  | int | *windowSize*, |
|  |  | int | *overlap* |
|  | ) |  |  |

static

|  |  |  |  |  |
| --- | --- | --- | --- | --- |
| boolean broad.core.sequence.WindowSlider.hasNext | ( |  | ) |  |

Here is the caller graph for this function:

|  |  |  |  |  |
| --- | --- | --- | --- | --- |
| SequenceRegion broad.core.sequence.WindowSlider.next | ( |  | ) |  |

|  |  |  |  |  |
| --- | --- | --- | --- | --- |
| void broad.core.sequence.WindowSlider.remove | ( |  | ) |  |

---

The documentation for this class was generated from the following file:

- java/broad/core/sequence/WindowSlider.java


---

Generated on Fri Aug 15 2014 15:10:56 for ESAT by  

 1.8.7
